# Association of the Fractal Dimension of Retinal Arteries and Veins with Quantitative Brain MRI Measures in HIV-Infected and Uninfected Women

**DOI:** 10.1371/journal.pone.0154858

**Published:** 2016-05-09

**Authors:** Howard A. Crystal, Susan Holman, Yvonne W. Lui, Alison E. Baird, Hua Yu, Ronald Klein, Diana Marcella Rojas-Soto, Deborah R. Gustafson, Glenn T. Stebbins

**Affiliations:** 1 Departments of Neurology, SUNY Downstate Medical School, Brooklyn, NY, United States of America; 2 Departments of Medicine, SUNY Downstate Medical School, Brooklyn, NY, United States of America; 3 Department of Radiology, NYU Langone School of Medicine, New York, NY, United States of America; 4 Department of Ophthalmology, University of Wisconsin School of Medicine, Madison, WI, United States of America; 5 Department of Neurological Sciences, Rush University School of Medicine, Chicago, IL, United States of America; Medical University of Graz, AUSTRIA

## Abstract

**Objective:**

The fractal dimension of retinal arteries and veins is a measure of the complexity of the vascular tree. We hypothesized that retinal fractal dimension would be associated with brain volume and white matter integrity in HIV-infected women.

**Design:**

Nested case-control within longitudinal cohort study.

**Methods:**

Women were recruited from the Brooklyn site of the Women’s Interagency HIV study (WIHS); 34 HIV-infected and 21 HIV-uninfected women with analyzable MRIs and retinal photographs were included. Fractal dimension was determined using the SIVA software program on skeletonized retinal images. The relationship between predictors (retinal vascular measures) and outcomes (quantitative MRI measures) were analyzed with linear regression models. All models included age, intracranial volume, and both arterial and venous fractal dimension. Some models were adjusted for blood pressure, race/ethnicity, and HIV-infection.

**Results:**

The women were 45.6 ± 7.3 years of age. Higher arterial dimension was associated with larger cortical volumes, but higher venous dimension was associated with smaller cortical volumes. In fully adjusted models, venous dimension was significantly associated with fractional anisotropy (standardized β = -0.41, p = 0.009) and total gray matter volume (β = -0.24, p = 0.03), and arterial dimension with mean diffusivity (β = -0.33,.p = 0.04) and fractional anisotropy (β = 0.34, p = 0.03). HIV-infection was not associated with any retinal or MRI measure.

**Conclusions:**

Higher venous fractal dimension was associated with smaller cortical volumes and lower fractional anisotropy, whereas higher arterial fractal dimension was associated with the opposite patterns. Longitudinal studies are needed to validate this finding.

## Introduction

Effective combination antiretroviral therapy (cART) has transformed HIV into a chronic illness and markedly decreased the prevalence of HIV-associated dementia [1]. Nonetheless, milder forms of cognitive impairment remain prevalent in patients treated with cART–even those with no detectable virus in their blood [1–2]. Many factors may contribute to cognitive impairment in such patients including brain vascular disease. Previous studies of the association between brain vascular health and cognition in HIV have focused on large vessels with ultrasound examination of the common carotid being the most frequent measure [3]. However, small vessel disease also contributes to brain dysfunction, and the relative severity of brain large and small vessel disease in any patient depends on many factors include age, race/ethnicity, and comorbid disease. For this reason, we hypothesized that measures of brain small vessel health would be associated with brain structure in patients with HIV.

Unfortunately, direct measurement of the caliber and branching patterns of brain small vessels is not possible with current technologies. However, retinal photographs offer a simple alternative. Retinal photographs have been used in epidemiological studies linking vascular health and cognitive outcomes for over 20 years [4–5]. Quantitative measures derived from these photographs include normalized arteriolar and venular width (i.e. total width, not luminal width) and more recently fractal dimension, a measure of the complexity of vascular tree. Previous quantitative retinal studies in persons infected with HIV have investigated arteriolar and venular width [6–10], but not fractal dimension. **T**o our knowledge, no previous studies have examined the association between these retinal measures and quantitative brain MRI measures in persons with HIV.

## Methods

### Cohort

Subjects were recruited from the Brooklyn Women’s Interagency HIV (WIHS) study site as part of a study of Vascular Health and Cognition in HIV. WIHS is a multi-center longitudinal study of HIV-infected and uninfected women that started in 1994. WIHS study visits occur every six months and include in-depth interviews, physical examinations and specimen collection; recruitment methods and data collection procedures have been described elsewhere [11–12]. For the current study, we recruited **a**ge-matched HIV-infected and uninfected in a 2: 1 ratio. This study was approved by our institutional review board (SUNY Downstate, approval #11–102, IRBnet ID number 266885–13) and all participants signed informed consent. Exclusion criteria included claustrophobia or metal implants, history of significant head injury, significant psychiatric disease, hepatitis C, ongoing substance abuse, and/or history of space occupying brain lesion. All subjects were proficient English speakers. Initially, 84 subjects received MRI scans; 10 were eliminated because of motion artefact.

### Imaging

MRI Imaging was done using a 3.0 Tesla Siemens Tim Trio MR scanner (Erlangen, Germany) using a standard 12 channel headcoil. MPRAGE (TE = 2.92 ms, TR = 2300 ms, flip angle = 9 degrees, in plane resolution = 1.1x1.1 mm, slice thickness = 1.2 mm) was performed with and without parallel imaging using GRAPPA factor 2. Axial FLAIR (TE = 81 ms, TR = 9000 ms, TI = 2500 ms, flip angle = 120 degrees, in plane resolution 1.0x0.7 mm, slice thickness = 5 mm, GRAPPA factor 2), and Diffusion EPI sequence (b = 0, 700, 64 directions, TE = 84 ms, TR = 7600 ms, FOV = 256x235 for in-plane resolution = 2.0x2.0 mm, slice thickness = 2 mm, GRAPPA factor 2) were also done.

MRI Image Processing: Post-acquisition processing of the MPRAGE images was performed using FreeSurfer v.5.3 software (http://surfer.nmr.mgh.harvard.edu/), using published methods [13–15]. Briefly, local cortical thickness measurements are based on differences between the position of equivalent vertices in pial and gray-white matter surfaces. Steps include white matter segmentation from T1-weighted images, estimation of gray-white matter interfaces, followed by examination for any geometric inaccuracies or topological defects remedied by automatic and manual corrections. Following this, deformable procedures lead to surface inflation of gray-white matter borders, spherical atlas registration, cortical gyral and sulcal structure parcellation, and creation of surface-based data maps [16]. Differences between subjects in gyral and sulcal depths are normalized; reconstructed brains of each subject are deformed and registered to an average spherical surface. Cortical and subcortical volumes were obtained from the FreeSurfer ver. 5.3 default subcortical segmentation routine Aseg [17].

Post-acquisition processing of diffusion tensor imaging (DTI) images utilized an open source suite of software developed at Stanford University (http://sirl.stanford.edu/software) with modifications developed in our laboratory (GS). Post-acquisition processing of DTI images first involved correction for eddy current distortions using a method developed by Rohde and colleagues [18] combining a rigid-body 3D motion correction (six parameters) with a constrained non-linear warping (eight parameters) based on a model of the expected eddy-current distortions. The DTI images were then co-registered to the MPRAGE structural images, using a preservation of principal diffusion direction algorithm to avoid distortion to the tensor directions during registration. The three eigenvalues (λ1, λ2 and λ3) and corresponding eigenvectors were calculated at each voxel, yielding measures of mean diffusivity (MD) fractional anisotropy (FA), axial diffusion (AD: λ1) and radial diffusion (RD: λ2 + λ3/2) according to the methods of Basser and Pierpaoli [19] and Song and colleagues [20–21]. Each individual DTI volume was examined with reference to the FLAIR image also co-registered the MPRAGE scan. If white matter hyperintensities were identified on the FLAIR image, manual editing of the DTI volumes was used to exclude the identified tissue. The white matter segment of the co-registered MPRAGE image was used as a mask for extraction of DTI parameters to give individual white matter estimates of MD, FA, AD and RD.

### Quantitative measures of retinal vasculature

Retinal photographs were obtained by a single investigator (HY) following established methods [22] using a Canon model CR2 digital non-mydriatic retinal camera (Canon USA Inc; Lake Success, NY 11042). A member of the University of Wisconsin Ocular Epidemioology Reading Center team trained and certified this investigator (HY).

Retinal artery central arteriolar and central venular equivalents (CRAE and CRVE) were determined at the University of Wisconsin Ocular Epidemiology Reading Center using the Interactive Vessel Analysis [IVAN]) software developed at the University of Wisconsin to measure the width of the erythrocyte column, which approximates the internal lumen diameters of retinal arterioles and venules[23]. Fractal dimension were determined at the University of Wisconsin Ocular Epidemiology Reading Center using the Singapore I Vessel Assessment (SIVA) program; a semi-automated computer assisted program developed by Tien Wong, Carol Cheung et al [24]. The software places a grid centered on the optic disc and identifies vessels as arteries or veins. The technician manually checks grid placement, vessel identification, vessel width, and vessel crossing, and makes corrections when necessary. The fractal value is calculated from skeletonized line tracing using the box-counting method, which divides each image into a series of squares for various side lengths [25–28]. The SIVA software determines fractal dimension within a range of 6 magnification levels relative to one unit of length in one direction of the image. Representative log-log plots have a linear pattern and have been published previously [28]. **In** a previous study evaluating SIVA software, the test-retest reliability between fractal determinations on the same photograph was 0.95; on different photographs of the same eye it was 0.46 [28]. Of the 88 women with retinal photographs, 12 cases were eliminated because of poor focus, an additional 4 were eliminated because of poor illumination leaving 72 with usuable fractal measurements. Values from the right and left eye were averaged.

### Final data set

Of the 74 women with usable MRIs and 72 with usable fractal measurements, 55 had a complete data set (34 HIV-infected and 21 HIV-uninfected) that comprised the group analyzed in this report. Those included in the study were younger (45.0 v 49.0, p = 0.03), and included more HIV-uninfected participants (38% v 17%, χ2 = 4.0, p = 0.04). The prevalence of AIDS was similar in those included (20%) and those not included (23%) in the study (χ2 = .16, p = 0.69). All but two on the HIV-infected persons were on cART; one of these 2 was a long term non-progressor.

### Statistics

Means between HIV-uninfected and HIV-infected participants were compared with the independent measures t-test. Because white matter intensities were severely positively skewed, we also analyzed the log of white matter intensities.

Because we had noted in preliminary analyses that the association of venous and arterial fractal dimension with brain outcome ran in opposite directions with increased arterial dimension associated with ‘increased cortical volume and increased venous dimension with decreased cortical volume, we systematically investigated the association of these measures in regression models. In the simplest models either arterial or venous dimension, but not both, were entered, and then we entered both atrial and venous dimension together. All models included age and intracranial volume and the retinal measure(s) of interest. Age and intracranial volume account for so much variance in cortical volumes that they were included even in the most basic models.

Other models were adjusted for systolic and diastolic pressure, ‘race/ethnicity’ and ‘HIV infected v. not HIV-infected.’ We recognize that other potential independent variables might have influence on these outcomes, but were limited by sample size.

Determination of ethnicity/race was self-selected. Most participants identified themselves as African-American, accordingly this variable was evaluated as ‘African-American’ or ‘not African-American.’

As this was a pilot study, significance was set at p < 0.05. All analyses were performed in IBM SPSS version 21.

## Results

Table 1 summarizes demographic, retinal, and MRI data in the study subjects divided into two groups based on their HIV-status. Only 2 possible differences were noted–a significant difference in arterial-venous ratio with higher values in the HIV-infected, and a nonsignificant difference in CRVE–with lower values in the HIV-infected.

**Table 1:** Demographic, retinal, and MRI measures in study participants. When p values were not listed they were at least > 0.4. WM–white matter, CRAE–central retinal arterial equivalents, CRVE–central retinal venous equivalents. FA–fractional anisotropy, MD–mean diffusivity, All fractal dimension values are dimensionless. A straight line has a fractal dimension of 1, and a plane has a fractal dimension of 2. FA and MD values range between 0 and 1 and are dimensionless. CRAE and CRVE are in microns. CRAE/CRVE is dimensionless. All volumes (total gray, subcortical volumes, and log of WM hyperintensities) are in milliliters. For continuous variables (i.e. all variables except number with hypertension, and number of African Americans) values listed are means with the standard deviation in parentheses. CD4 number is cells per microliter.

	HIV uninfected (N = 21)	HIV-infected (N = 34)
Age	45.7 (6.9);	45.6 (7.4)
Number African American/ total number subjects	18/21	25/34
Number with hypertension	8/21	11/34
Current CD4 Number	NA	574 (237)
Overall fractal dimension	1.46 (0.04)	1.47 (0.04)
Arterial fractal dimension	1.25 (0.04)	1.26 (0.04)
Venous fractal dimension	1.25 (0.04)	1.24 (0.04)
CRAE (microns)	155 (11)	158(15)
CRVE	236 (19)	227 (22), p = 0.12
CRAE/CRVE	.66 (.03)	.69 (0.05), p = 0.002
FA	.363 (0.025)	0.361 (0.024)
MD	0.800 (0.044)	0.802 (0.035)
Total gray volume (ml)	516 (51)	518 (50)
Subcortical volume (ml)	52.1 (5.4)	51.2 (4.9)
Log WM hyperintensities	3.2 (0.1)	3.2 (0.2)

In regression models, we systematically evaluated the associations of arterial fractal dimension, or venous fractal dimension, or both arterial and venous fractal dimension, with brain outcome. Table 2 illustrates these analyses for the outcome of fractional anisotropy (FA), but the results were similar for all of the brain outcomes. When the models included either arterial or venous fractal dimension but not both, the associations were never significant in any model. When both arterial and venous fractal dimension were included, the absolute values of the beta coefficients increased, and significance improved.

**Table 2:** Regression models of the association of fractal dimension with fractional anisotropy. The predictors included in the model are listed in the first column, the second column lists the predictor of interest, and the third column lists the standardized β coefficients and p values. ICV = intracranial volume. Fractal anisotropy (FA) was the dependent variable for all analyses in the table. The standardized beta coefficient for arterial dimension is this model was 0.17, p = 0.21.

All included predictors	Predictor of interest	Beta, p
Age, ICV, arterial dimension	Arterial dimension	0.17,.0.21
Age, ICV, venous dimension	Venous dimension	-0.25,.07
Age, ICV, arterial, & venous dimension	Arterial dimension	0.35,.02
Age, ICV, arterial, & venous dimension	Venous dimension	-.41,.006
Age, ICV, systolic blood pressure, diastolic blood pressure, arterial dimension	Arterial dimension	0.15,.28
Age, ICV, systolic blood pressure, diastolic blood pressure, venous dimension	Venous dimension	-0.22,.0.12
Age, ICV, systolic blood pressure, diastolic blood pressure, arterial dimension, venous dimension	Arterial	0.34,.0.03
Age, ICV, systolic blood pressure, diastolic blood pressure, arterial dimension, venous dimension	Venous	-0.39, 0.01

The associations of retinal arterial and venous fractal dimension with the 6 MRI outcomes are shown in Table 3. Most strikingly, without exception, the associations were always in the opposite direction. For example, higher arterial fractal dimension was significantly associated with increased FA, but higher venous fractal dimension was significantly associated with *decreased* FA. In models only adjusted for age and intracranial volumes, these associations were significant for 7 of 10 outcomes: arterial dimension with FA, MD, the log of white matter hyperintensities, or with subcortical volumes; venous dimension with FA, gray volume, or with the log of WM hyperintensitiesand marginal in an eighth -venous dimension with subcortical volume. In fully adjusted models that included systolic and diastolic pressures, presence or absence of HIV-infection, and ethnicity as cofactors, 4 of 10 associations remained significant: arterial dimension with FA or with MD; venous dimension with FA or with total gray volume. Two more were of marginal significance: venous dimension with the log of WM hyperintensities (β = 0.27, p = 0.09) and arterial dimension with subcortical volume (β = 0.19, p = 0.06).

**Table 3:** Associations of arterial and venous retinal fractal dimension with brain outcomes. N = 55. Values listed are standardized β-coefficients and significance levels from linear regression models. All models included both arterial and venous dimension in addition to the variables listed in the top row. AA–African-American race/ethnicity, HIV–HIV-infected, ICV–intracranial volume. FA–fractional anisotropy, MD–mean diffusivity, WM–white matter.

Outcome	Predictors	Model 1; adjusted for age, ICV	Model 2; Adjusted for age, ICV, systolic, diastolic, AA, HIV
Log WM hyperintensities	Arterial dimension	-0.30, .05	-.25,.12
	Venous dimension	0.33,.03	0.27,.09
FA	Arterial dimension	.35, .02	0.34,.03
	Venous dimension	-.41, 0.006	-0.41,.009
MD	Arterial dimension	-.34, 03	-.33,.04
	Venous dimension	.24,0.13	0.23,.17
Total gray volume	Arterial dimension	.16,.11	.11,.29
	Venous dimension	-.27,.01	-.24,.03
Subcortical volume	Arterial dimension	0.21,.02	.19,.06
	Venous dimension	-.16,.09	-.16,.12

In contrast to fractal dimension, the association of central retinal arterial equivalents (CRAE) and/or central retinal venous equivalents (CRVE) with brain outcomes was only of marginal significance in one out of 10 possible outcomes. The standardized β for CRAE with the log of abnormal white matter intensities was = -0.44, p = 0.06 in fully adjusted models. None of the other relationships with CRAE and CRVE and brain outcomes approached statistical significance.

To explore the interactions between fractal dimensions, age, and blood pressure in more detail, we also performed linear regression analyses where fractal dimension was the outcome variable. As demonstrated in Table 4, age was strongly negatively associated with venous dimension (standardized β = -0.43, p = 0.002), but the association with arterial dimension was more modest (standardized β = -0.17, p = 0.23).

**Table 4:** Association of age and blood pressure with fractal dimension. Values listed are standardized beta coefficients and significance levels for linear regression models predicting either mean arterial dimension or mean venous dimension where age, systolic, and diastolic blood pressures were independent variables.

OUTCOME	Predictors	Standardized beta, p
Mean arterial dimension	Age	-.17,.24
	Systolic	-0.006,.97
	Diastolic	-.22,.20
Mean venous dimension	Age	-.40,.007
	Systolic	.12,.49
	Diastolic	-0.03,.86

## Discussion

To our knowledge, there are 3 unique features of this report: We are the first: 1) to relate estimations of fractal dimension to quantitative brain MRI measures in persons with HIV; 2) to suggest that the associations of retinal arterioles and venules fractal dimensions with brain MRI outcomes are in opposite directions with increasing arterial dimension associated with increasing FA and increasing venous dimension with decreasing FA; and 3) to demonstrate that the negative association of fractal dimension with age is largely due to venous fractal dimension.

We are aware of only 2 previous studies of the relationship between retinal fractal dimension and brain outcomes. Hilal et al [29] investigated the association of fractal dimension with white matter lesion volume (among other outcomes) in 261 Chinese patients whose mean age was 70. They also found that the association of arterial and venous fractal dimension ran in opposite directions with increasing venous dimension associated with the ‘worse’ outcome. However, the associations were not statistically significant.

Cavallari, Falco et al. [30] looked at 10 patients with cerebral autosomal dominant arteriopathy with subcortical infarcts and leukoencephalopathy (CADASIL) and 10 age-matched controls. The CADASIL patients had lower overall (arterial and venous) fractal dimension, but they found no correlation between the very low levels of white matter lesion volume and overall fractal dimension. They examined overall fractal dimension rather than including both arterial and venous dimension in their models. Fractal analyses have also been used in pathological studies; examples include quantification of plaques in Alzheimer disease brains [31] and astrocyte subtypes in stroke and dementia [32].

Whereas we are the first to emphasize that the association of arterial and venular fractal dimension with MRI measures track in opposite directions, this pattern has been demonstrated in studies of normalized retinal arteriolar and venular diameters. In these studies, increasing venular diameters have been associated with worse cognition [30] and increased cortical atrophy. On the other hand, decreasing arteriolar diameters have been associated with impaired white matter integrity [33–34] increased mean diffusivity, and decreased cortical volume.

We hypothesize that at least 3 general disease processes contribute to brain changes in our participants: ischemia, inflammation, and circulatory system pressure changes leading to changes in pressures in capillaries and venules as well as interstitial spaces of the brain. The finding that higher arterial dimension tended to have less brain atrophy is intuitively attractive to the extent that higher dimension may mean that tissue is more likely to receive adequate oxygen and glucose. We acknowledge that the relationship is probably more complex than linear, and that both excessively high and excessively low arterial fractal dimension may be associated with worse outcomes. For example, Liew et al [35] showed that the middle 2 quartiles of fractal dimension were associated with the best cardiovascular outcomes. The Liew study used the a different software package (IRIS-FRACTAL) than the SIVA software used in our study. However, both methods use skeletonization and box counting. The mean D_f_ in their study that included 3303 men and women with a mean age of 65 (8.6) was 1.44 (0.024), similar to our finding of a D_f_ of 1.46 (0.0371).

On the other hand, the observed association of increased venous dimension with smaller cortical volumes might be explained if increased venous fractal dimension is a surrogate for increased inflammation and/or suboptimal intravascular/interstitial pressure gradients. Empirical data could be used to explore the association between systemic inflammation and venous fractal dimension. The effects of intravascular/interstitial pressure gradients could be investigated with animal models. Increased venous fractal dimension could simultaneously be associated with decreased ischemia and with increased inflammation. This hypothesis would explain the regression findings of the association of arterial and venous fractal dimension with brain structure running in opposite directions. Variance in structural measures is probably better explained by independent variables that are surrogates for ‘ischemia’ and ‘inflammation’ than by models that include only ischemia (perhaps arterial fractal dimension) or by a variable where the effects simultaneously run in opposite directions (increased venous dimension may be a marker of increased inflammation and decreased ischemia–relatively cancelling each other out). Perhaps when both arterial and fractal dimension are included in the models, arterial dimension becomes largely a surrogate for ischemia and venous dimension largely a surrogate for inflammation. Such reasoning would also begin to explain the apparently contradictory effects of age on brain structure and venous dimension.

Several previous studies have investigated the association of age and retinal fractal dimension. Doubal et al [36] reported a negative association with age, but did not specify different associations for arterioles and venules. Azemin, Kumar et al. [37–38] also found that fractal dimension significantly decreased with age, but again did not look at arterioles and venules separately. Although cross-sectional studies suggest that fractal dimension declines with age, this needs to be confirmed in longitudinal studies.

A strength of this study is the ability to relate fractal dimension of retinal arterioles and venules to multiple quantitative brain outcomes. Weaknesses include moderate sample size with only 62% o**f** participants with a complete dataset.

Focal areas of increased signal in the white matter on FLAIR and T2-weighted brain MRIs are prevalent in multiple cohorts and increase with aging. This finding is detected on thousands of brain MRIs around the world each day, and in persons over age 50 are almost always attributed to ‘ischemic microvascular disease’ unless some known other pathological process such as multiple sclerosis has been previously documented. Nonetheless, the presumption of ‘ischemic microvascular disease’ is an inference based on radiologic-pathological studies. Ideally, one would like to directly assess brain small vessel anatomy in these cases. Until noninvasive methods to analyze brain small vessel health are perfected, retinal photographs offer an alternative.

## Supporting Information

S1 Dataset PLOS_ID–arbitrary de-identified ID number with no personal information; CD4 number–cells per microliter; CRAE–central retinal arterial equivalents (in microns), CRVE–central retinal venous equivalents (in microns), CRAE/CRVE central retinal equivalents A/V ratio, MD—mean diffusivity (of right and left cerebral hemisphere white matter), FA–fractional anisotropy (of right and left cerebral hemisphere white matter), AA–self identified as of African ethnicity, WM hyperintensities–volume of white matter hyperintensities in ml, SubCortGrayVol—Volume of subcortical gray matter in ml, TotalGrayVol–gray matter volume of right and left cerebral hemispheres (cortical and subcortical) in ml, ANYHTN–any hypertension as defined in text, age–age when MRI obtained in years, logWM–log (base 10) of white matter hyperintensities, ICV–intracranial volume in ml, HIV_infected (1: participant is HIV-infected, 0: participant is not HIV-infected). (XLSX)
